# Developing a Multi-Element Sensor to Non-Destructively Monitor Several Fundamental Parameters Related to Concrete Durability

**DOI:** 10.3390/s20195607

**Published:** 2020-09-30

**Authors:** Ming Jin, Yuefeng Ma, Haoyu Zeng, Jiaping Liu, Linhua Jiang, Guo Yang, Yue Gu

**Affiliations:** 1 School of Material Science and Engineering, Southeast University, Nanjing 211189, China; jinming@seu.edu.cn (M.J.); zenghaoyu1511@foxmail.com (H.Z.); 230189610@seu.edu.cn (G.Y.); 2 State Key Laboratory of High Performance Civil Engineering Material, Nanjing 210008, China; 201909021028@cqu.edu.cn; 3 College of Materials Science and Engineering, Chongqing University, Chongqing 400045, China; 4 College of Mechanics and Materials, Hohai University, Nanjing 210098, China; lhjiang@hhu.edu.cn (L.J.); Gubetter@hhu.edu.cn (Y.G.); 5 National Engineering Research Center of Water Resources Efficient Utilization and Engineering Safety, Nanjing 210008, China

**Keywords:** monitoring, sensor, chloride, freezing-thawing, concrete

## Abstract

A design scheme of multi-element sensor which included electrical resistivity probes, multiple Cl^−^ selective electrodes, and a steel corrosion monitoring system was proposed in this work. Embedding this multi-element sensor in concrete enables the real-time and non-destructive monitoring of internal electrical resistivity, free Cl^−^ (Cl_f_) contents in the concrete pore solution at different depths, and steel corrosion parameters. Based on the monitoring data obtained by the multi-element sensor, the freezing-thawing (F-T) damage degree, the Cl_f_ diffusion coefficient, the quantitative relation between F-T damage degree and Cl_f_ diffusion coefficient, the initiation period of steel corrosion, and the critical content related to steel corrosion are determined. To conclude, the multi-element sensor provides key durability parameters for the establishment of the Cl_f_ diffusion model, the assessment of health condition, and the prediction of service life of concrete under the coexistence of the F-T cycle and Cl^−^.

## 1. Introduction

Modern reinforced concrete is widely applied due to its excellent workability, mechanical properties, and potential durability [1]. Reinforced concrete durability has attracted great attention especially in marine areas where reinforced steel in concrete faces severe corrosion. Passive film adhered to the steel surface becomes unstable and is then destroyed when the content of chloride ions (Cl^−^) around steel reaches a threshold value [2]. The single Cl^−^ penetration mechanism and the impact of Cl^−^ on steel corrosion have been widely reported in previous research [3,4]. However, this is inconsistent with the real service environment where concrete suffers from more than one environmental factor, such as the F-T cycle, carbonation, drying-wetting cycle, and thermal gradient. The coupled action of Cl^−^ and environmental factors usually leads to more severe deterioration and complex damage mechanisms than the single Cl^−^ works [5,6,7]. Specifically, in the marine and cold coexisting region, the F-T cycle leads to the generation and propagation of microcracks in concrete which, in turn, accelerates the Cl^−^ diffusion rate and increases Cl^−^ penetration depth [8,9] in concrete. Consequently, the F-T cycle increases the risk of initiating steel corrosion and the corrosion rate of steel [10]. The premature or unexpected failure of concrete due to steel corrosion or F-T damage not only affect the structural integrity and reduce the service life of the concrete structure, but also leads to high maintenance and repair costs.

On-demand maintenance of concrete structures ensures construction safety, prolongs the service life, and avoids further financial loss. For a scheduled and preventive maintenance program in concrete structure, the continuous and in-situ monitoring of parameters related to concrete durability is crucial [11,12,13]. Refs [14,15] reviewed numerous existing non-destructive measurement techniques of steel corrosion in concrete. Among them, electrochemical techniques are most appropriate to non-destructively and in-situ monitor steel corrosion conditions in concrete structures due to the following reasons: (i) steel corrosion is electrochemical in nature, and (ii) easily operated and costly equipment for electrochemical measurement [14,15,16]. More reliable and accurate corrosion results of steel in concrete could be obtained through the embeddable reference electrodes [17] because the errors produced by the membrane potential and diffusion potential could be minimized [18]. In the authors’ previous research [19,20], solid Ag/AgCl reference electrodes have been developed and this kind of sensor was embedded in concrete to conduct electrochemical measurements of Cl^−^ induced steel corrosion. However, the reliability of monitoring steel corrosion parameters using embeddable Ag/AgCl reference electrode in concrete still needs to be confirmed in the condition of F-T cycle.

Refs [21,22] reviewed tens of non-destructive measurement techniques of Cl^−^ content in concrete such as potentiometric measurement, electrical resistivity, impedance analysis, fiber optic sensor, laser breakdown spectroscopy, nuclear magnetic resonance, and X-ray diffraction analysis. However, most of them were limited to be used in the laboratory and unable to provide accurate information about the free Cl^−^ (Cl_f_) content in pore solution of concrete. Compared to other techniques, the potentiometric technique such as Ag/AgCl sensor is more appropriate conduct non-destructive and in-situ measurement of Cl_f_ concentration due to these advantages such as accuracy and simplicity [23]. As early as the 1990s, Atkins et al. [24] and Climent-Llorca et al. [25] reported using the Ag/AgCl sensor to detect the Cl_f_ concentration in simulated pore solution and mortar specimens. Elsener et al. [26] found out that the calibration relation between the potential of Ag/AgCl sensor and Cl_f_ concentration in synthetic concrete pore solution followed Nernst law. Furthermore, the Cl_f_ content determined by the Ag/AgCl sensor correlated well with that by pore solution expression. Angst et al. [18] suggested that the diffusion potential between the reference electrode and Ag/AgCl sensor influenced the accuracy of measuring results and therefore the reference electrode should be positioned as close to the Ag/AgCl sensor as possible in order to reduce these errors. Montemor et al. [27] have embedded multiple Ag/AgCl sensors in different depths of mortar samples to monitor the Cl_f_ profiles and concluded that the Cl_f_ diffusion in mortar specimens followed the Fick’s law. Although a large amount of research has applied this type of potentiometric sensor to monitor the Cl_f_ content in mortar paste and concrete, determining the critical Cl^−^ content of steel corrosion in concrete as an expression of Cl_f_ is scare. The critical Cl_f_ value related to the initiation of steel corrosion in concrete could be obtained by simultaneously monitoring the steel corrosion parameters and Cl_f_ content in the vicinity of steel in concrete using the embeddable reference electrode and Ag/AgCl sensor.

For concrete structures in the cold region, a freezing-thawing (F-T) cycle is usually the primary cause of the deterioration of concrete. Currently, widely used methods of characterizing the damage degree of concrete caused by F-T cycle are indirect techniques such as dynamic elastic modulus [28], mechanical strengths [29] and transport properties [30]. However, these indirect techniques are suitable in laboratory conditions and not applicable to detect F-T damage degree of the in-situ concrete structure. Recently, ultrasonic imaging [31,32], X-ray computed tomography, and acoustic emission techniques [33,34] have been applied to non-destructively and quantitatively estimate the deterioration degree of mortar and concrete after being subjected to F-T cycle by detecting the change on the microstructure. Although ultrasonic imaging and X-ray computed tomography implement the visualization of the microstructure of mortar and concrete samples and detects the generation of crack, none of them might be suitable to real-time monitor and evaluate the F-T deterioration degree of field concrete structure. Chung et al. [35] pointed out that the increase or decrease in temperature during the F-T cycle changed electrical resistivity of concrete irreversibly while F-T damage increased electrical resistivity reversibly. Nevertheless, Ding et al. [36] argued that the F-T cycle reduced the electrical impedance of concrete and measurement of electrical impedance of concrete was a non-destructive way of assessing the frost damage on concrete. Farnam et al. [37] suggested that the variation on the electrical resistivity of mortar samples after F-T cycle was attributed to the cracking generated on the microstructure. The F-T cycle damages the microstructure of concrete and thereby changes the electrical resistivity because the electrical resistivity of concrete is dependent on the electrical resistivity of concrete pore solution and internal microstructure with the assumption that F-T cycle has no influence on the electrical resistivity of concrete pore solution. In refs [35,36], the method of measuring electrical resistivity through surface wrapped electrodes is only suitable for samples casted in the laboratory and fails in monitoring the electrical resistivity of filed concrete construction. Thus, the real-time and non-destructive measurement of electrical resistivity of field concrete structure demands an embeddable electrical resistivity sensor with which the F-T damage degree could be accurately determined.

Currently, there lacks a multifunctional sensor to simultaneously detect corrosion condition of steel and Cl_f_ content in concrete pore solution and F-T damage degree of concrete. This paper aims to provide a design method of multi-element sensor which includes electrical resistivity probes, multiple Cl^−^ selective electrodes, a carbon steel electrode, an embedded solid reference electrode, and a counter electrode. Then, embedding the multi-element sensor in concrete to monitor the internal electrical resistivity and Cl_f_ contents at different depths of concrete and corrosion potential as well as corrosion rate of steel in concrete. As a result, parameters closely related to concrete durability, such as F-T damage degree, Cl_f_ diffusion coefficient, and threshold value of steel corrosion were determined. This provides an essential reference for the prediction of the residual service life of a concrete structure, especially in a coexisting environment of F-T cycle and Cl^−^.

## 2. Fabrication and Monitoring Principle of the Multielement Sensor

### 2.1. Preparation of Multielement Sensor

**Figure 1:**
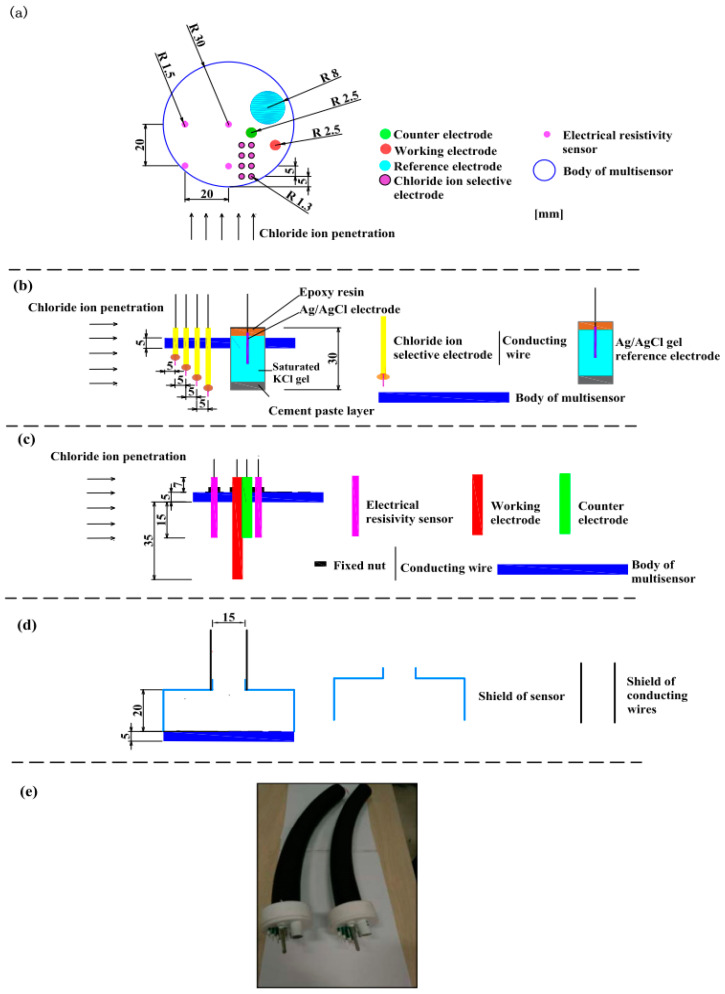
The schematic diagram and photograph of the multielement sensor. (**a**) top view of sensor, the location and size of the drilled holes for every kind of electrode, (**b**) side view of sensor, placement of Cl^−^ selective electrodes and reference electrode, (**c**) side view of sensor, placement of electrical resistivity probes, counter electrode and working electrode, (**d**) placement of cylindrical shield and cable protection sleeve, and (**e**) the photo of prepared multielement sensor.

**Figure 2:**
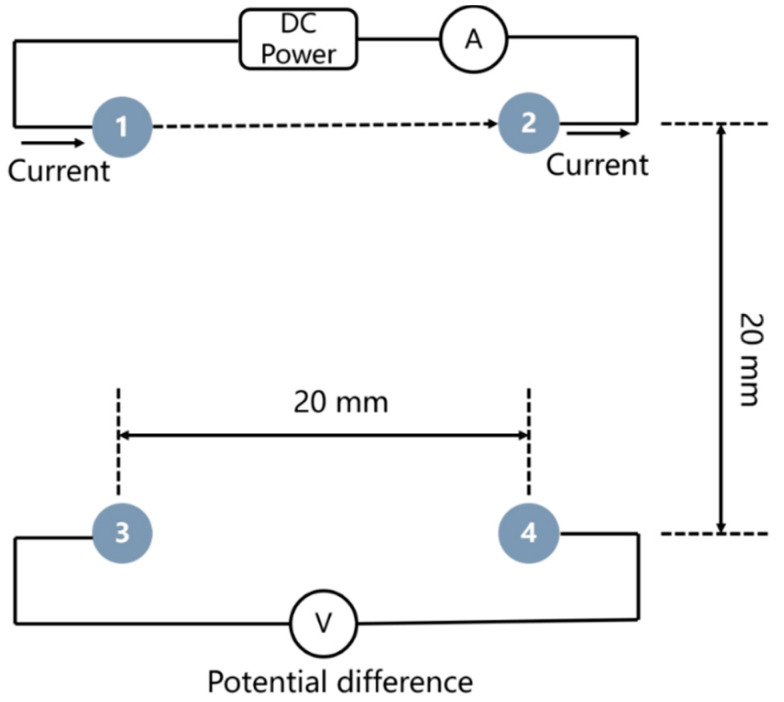
The schematic diagram of measuring the electrical resistivity of internal concrete using electrical resistivity probes.

The multielement sensor included electrical resistivity probes, multiple Cl^−^ selective electrodes, a carbon steel (working electrode), a solid Ag/AgCl reference electrode and a stainless-steel electrode (counter electrode). Figure 1 indicates the schematic diagram of fabricating the multielement sensor: (1) PVC disk (60 mm in diameter and 5 mm in thickness) was selected as the body of the multielement sensor where holes were drilled so that every kind of sensor could be fixed, as shown in Figure 1a; (2) Cl^−^ selective electrodes with different lengths and a solid Ag/AgCl reference electrode containing saturated KCl gel electrolyte were inserted into corresponding holes (as shown in Figure 1b), the detailed fabrication process of Cl^−^ selective electrode and Ag/AgCl electrode was introduced in our previous research [20,38]; (3) electrical resistivity probes, working electrode and counter electrode were mounted in the body of sensor and fixed using plastic nuts, as shown in Figure 1c; (4) a cylindrical shield (60 mm in diameter and 20 mm in height) was applied to cover the upper surface of the multielement sensor and isolated the connections between electrodes and conducting wires from external environment, then a cable protection sleeve was connected tightly to the shield to protect the conducting wires, as shown in Figure 1d; (5) all the connections on the surface of the sensor were sealed with sealant to prevent external liquid from entering into the sensor, the photo of prepared multielement sensors is shown in Figure 1e.

### 2.2. The Multi Functions of the Prepared Multielement Sensor

#### 2.2.1. Monitoring the Cl_f_ Content in Concrete Pore Solution

Cl_f_ content in concrete was monitored through the measurement of Cl^−^ selective electrode potential because there existed a linear response between the logarithmic value of Cl_f_ content in pore solution and Cl^−^ selective electrode potential. The potential of the Cl^−^ selective electrode was directly recorded using the voltmeter with respect to the embedded reference electrode. Based on our previous research [7], the response equation between Cl^−^ selective electrode potential and Cl_f_ content depended on the alkalinity of pore solution and therefore three rules were set in advance for calculating Cl_f_ content using electrode potential: (1) the response equation at pH of 13.5 was used once the pH of pore solution exceeded 13, (2) the equation at pH value of 12.5 was used when the pH value of pore solution was between 11 and 13, and (3) the equation at pH of 10 was used when the pH of pore solution was lower than 11.

The pH of concrete powder around Cl^−^ selective electrode (distance <2 mm) was determined using the solid-liquid extraction method. Firstly, concrete power (excluding coarse aggregate) around Cl^−^ selective electrode (distance <2 mm) was collected, ground, and then passed through a 0.16-mm sieve. Subsequently, 1 g of powder was soaked in 10 g of deionized water and the mixture was stood for 24 h after a powerful stirring. Then, the mixture was filtered, and the pH of filtrate was recorded by pH meter.

#### 2.2.2. Monitoring the Electrical Resistivity of Internal Concrete

Figure 2 illustrates the principle of measuring the electrical resistivity of internal concrete using the electrical resistivity probes arranged in a square with a side length of 20 mm. The two electrodes on the same side (electrode named as No. 1 and 2 in Figure 2) were passing a known input current (*I*, approximately 0.5 mA) and potential difference (Δ*V*) between the other two electrodes (No. 3 and 4 in Figure 2) was recorded. The electrical resistivity of concrete was calculated by introducing the geometrical factor (*GF*) [39], as expressed in Equation (1):

(1) $$\rho \;=\;GF\frac{∆V}{I}$$

(2) $$GF\;=\;\frac{2\pi a}{2\;-\;\sqrt{2}}$$

where a is the side length of the square (20 mm in this study).

#### 2.2.3. Monitoring Steel Corrosion in Concrete

The potential of carbon steel electrode was measured against the reference electrode using a voltmeter. In addition, the electrochemical impedance spectroscopy (EIS) measurement was performed by a three-electrode system in the frequency between 100 kHz and 0.1 Hz using a potentiostat (PARSTAT 2273, Princeton Applied Research, Oak Ridge, TN, USA).

## 3. Materials and Methods

### 3.1. Materials

Ordinary Portland cement and grade II fly ash were used and their oxide compositions are listed in Table 1. Local river sand (fineness modulus is 2.6) and gravel (size between 5 mm and 20 mm) were used. Commercial polycarboxylate high performance water reducing agent was used to modify the working performance of concrete and air entraining agent was applied to entrain air in concrete. The chemical composition (wt.%) of carbon steel in the multielement sensor is listed in the following: C (0.22%), Si (0.31%), Mn (0.64%), S (0.05%), P (0.05%) and the residual Fe. Distilled water was used, and all other reagents were analytically pure.

### 3.2. Mixture Proportions of Concrete and Concrete Specimens

Table 2 lists the mixture proportions of concrete. 0.5 was chosen as the ratio of water to binder. 40% of cement was replaced with fly ash in FC concrete and air entraining agent was added in AC concrete. Fly ash and air entraining agent were simultaneously used in FAC concrete.

**Figure 3:**
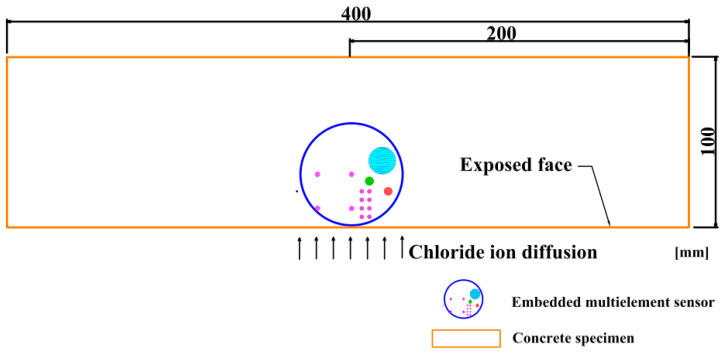
The schematic diagram of concrete specimen embedded with a multielement sensor.

The 100 × 100 × 400 mm rectangular concrete specimens were casted. During the casting, the multielement sensor was placed in concrete and the diagram of concrete specimen containing sensor is indicated in Figure 3. The concrete specimens were demolded after 24 h and then cured in the curing room (20 ± 1 °C and 95 ± 2% RH) for 4 weeks. The air content of fresh mixed concrete was measured using a pressure meter following the Chinese standard GB/T 50081-2002 [40] and the compressive strength of concrete (without sensor) after curing period of 28 days was recorded. Then, 5 groups of fresh mixed concrete and 5 concrete specimens were applied to obtain the average results of air content and compressive strength.

### 3.3. Exposure to F-T Cycle and Subsequent Cl^−^ Penetration

**Figure 4:**
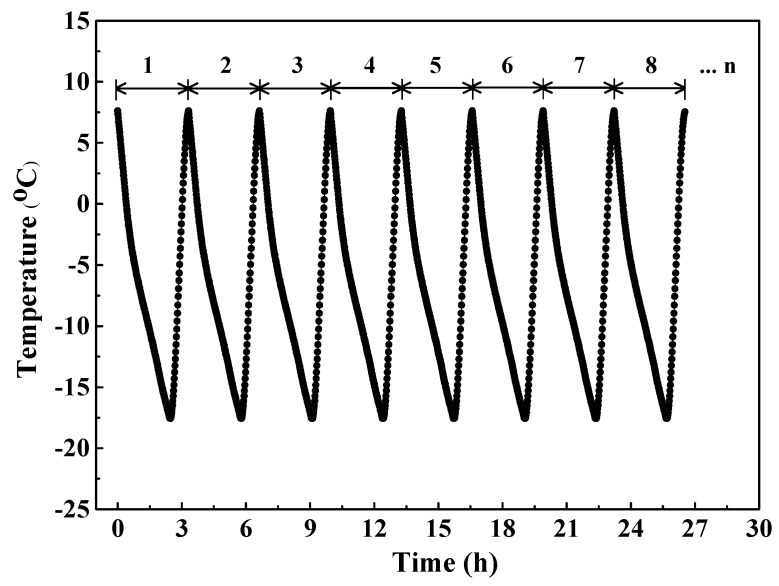
The development of concrete temperature during the process of F-T cycle.

After 28-day curing, concrete was subjected to the “rapid F/T testing method” introduced in the Chinese standard GB/T 50082–2009 [41]. Firstly, concrete specimens were fully immersed in water for 4 d to ensure the sufficient saturation condition; secondly, specimens were stored in stainless steel boxes and subsequently water was added into the stainless steel boxes until the water level was 5 mm higher than the top face of concrete specimen; then the boxes were placed in the rapid F/T machine (Suzhou Donghua Examination Appartus Co., Ltd. Suzhou, China) to conduct F-T experiment. In every cycle, the concrete temperature dropped from 7.5 °C to −17.5 °C with an temperature reducing rate of approximately 10 °C/h and increased from −17.5 °C to 7.5 °C with an temperature increasing rate of approximately 25 °C/h. Figure 4 shows the evolution of concrete temperature with time in the procedure of F-T cycle. During the 1st, 25th, 75th, and 125th F-T cycles, the electrical resistivity of internal concrete was continuously monitored and recorded. Three concrete specimens were used to obtain the average results. Furthermore, the relative dynamic modulus measurement was performed following ASTM C597-2002 [42] after 1, 25, 75, and 125 F-T cycles and this testing was also conducted on 3 concrete specimens to obtain the average value.

After exposure to 1, 25, 75, and 125 F-T cycles, five faces of concrete specimens were totally covered with epoxy resin and leaving one of side faces unsealed. Then concrete was soaked in 1 mol/L NaCl solution which was updated every two weeks. The Cl^−^ selective electrode potential was measured vs. solid Ag/AgCl reference electrode and recorded in a total immersion period of 36 weeks. In addition, half-cell potential and electrochemical impedance spectroscopy of carbon steel were also operated during the immersion. 5 concrete specimens were used to obtain the average results.

## 4. Results and Discussion

### 4.1. Monitoring F-T Damage on Concrete by Monitoring Electrical Resistivity

**Figure 5:**
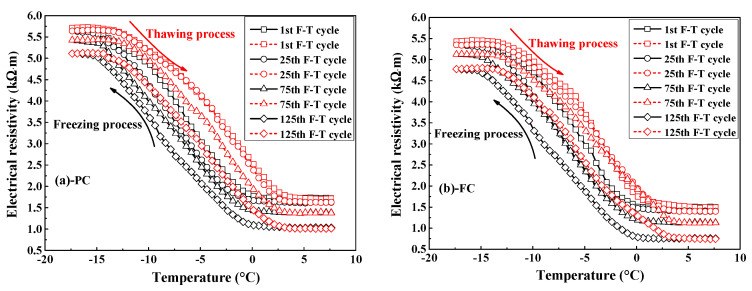

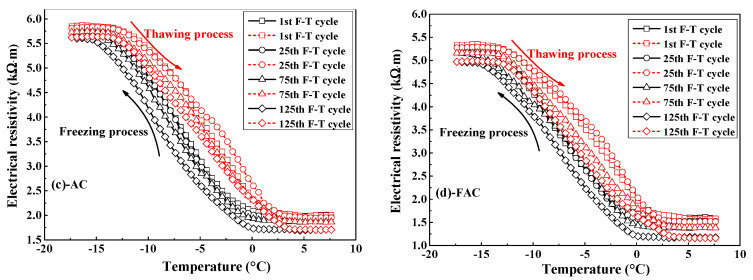
Typical plots of electrical resistivity of various concrete types against the temperature at different times of F-T cycle: (**a**) PC type, (**b**) FC type, (**c**) AC type, and (**d**) FAC type.

Figure 5 indicates the electrical resistivity of concrete versus the temperature at 1st, 25th, 75th, and 125th F-T cycles. In the freezing stage, the electrical resistivity of concrete keeps the initial value when temperature is higher than 0 °C and the electrical resistivity quickly increases with the decrease in temperature due to the ice formation [37] since the electrical resistivity of ice is greater than pore solution [43]. Finally, the electrical resistivity of concrete reaches maximum value because the most volume of pore solution in concrete has been changed into ice. In the thawing stage, the electrical resistivity of concrete firstly keeps the maximum value and then drops quickly with temperature due to the ice melting, the electrical resistivity of concrete reaches minimum value when this part of ice has been totally melted into liquid. The temperature-dependence characteristic of electrical resistivity of concrete has also been reported in refs [37,44]. Differing from the measurement of electrical resistivity of whole mortar or concrete specimens in refs [37,44], the multi-element sensor developed in this work is capable of continuously monitoring the electrical resistivity of internal concrete in the process of the F-T cycle.

**Figure 6:**
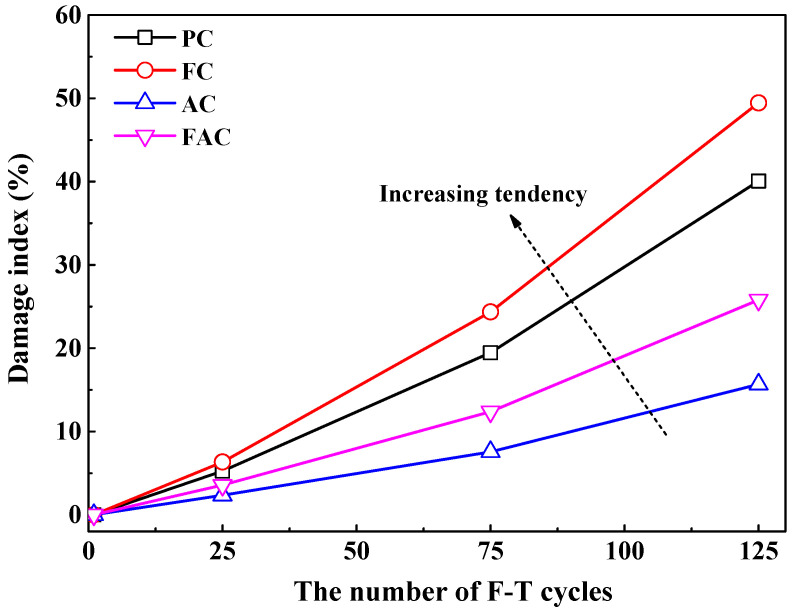
The damage index as a function of the number of F-T cycles.

The electrical resistivity results indicate that the electrical resistivity of concrete at the end of the thawing stage decreases with F-T cycle. According to ref [45], the electrical resistivity of concrete depends on the electrical resistivity of pore solution, porosity and connectivity of pores in concrete, as expressed by Equation (3):

(3) $$\rho =\rho_{o}\frac{1}{ø\beta }=\rho_{o}\left(FF\right).$$

in which ρ represents the electrical resistivity of concrete, $\rho_{o}$ represents the electrical resistivity of concrete pore solution, ø represents the total porosity of concrete and β represents the connectivity of pores in concrete. Assuming no change in the pore solution after F-T cycle, any variation in the electrical resistivity of concrete after exposure to F-T cycle is attributed to the change in this term of $\frac{1}{ø\beta }$, usually known as “formation factor” (FF) [46]. The F-T cycle reduces the formation factor of concrete because damage caused by F-T cycle increases the porosity [47] and connectivity of pore system in concrete. The reduction on formation factor of concrete due to F-T cycle has also been reported in ref [37]: the more serious F-T damage, the larger reduction on the formation factor of concrete. Consequently, the variation of the formation factor is closely related to the deterioration on concrete due to F-T cycle and thereby the change on the formation factor represents the damage index for concrete after F-T cycle, as shown in Equation (4):

(4) $${\left(Damage\;index\right)}_{n}\;=\;\left(1\;-\;\frac{{FF}_{n}}{{FF}_{1}}\right)\;\times \;100\%\;=\;\left(1\;-\;\frac{\rho_{n}}{\rho_{1}}\right)\;\times \;100\%$$

where n and 1 represent n and 1 F-T cycles respectively. Generally, the damage index increases with the increasing number of F-T cycles, as shown in Figure 6. Compared with plain cement concrete, concrete added with fly ash has a higher damage index, but concrete supplemented with air entraining agent has a lower damage index, revealing that the addition of fly ash reduces while air entraining age improves concrete’s ability of resisting F-T deterioration. Furthermore, the improvement on concrete resistance to F-T damage by air entraining agent is stronger than the reduction on concrete resistance by fly ash since concrete with joint addition of air entraining agent and fly ash has lower damage index compared with plain cement concrete.

**Figure 7:**
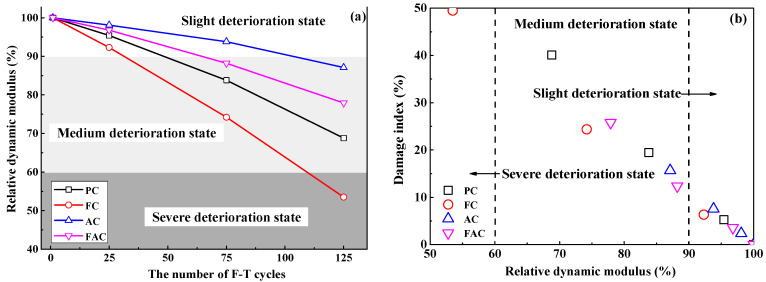
(**a**) the development of relative dynamic modulus with the number of F-T cycles and (**b**) the damage index as a function of relative dynamic modulus.

The relative dynamic modulus of concrete has been widely used to characterize the F-T damage degree [9,48] and ref [28] proposed a methodology for qualitatively assessing the deterioration degree of concrete after F-T cycle based on the relative dynamic modulus results. Figure 7a shows the development of relative dynamic modulus of prepared concrete with F-T cycles; furthermore, Figure 7b indicates the damage index as a function of the relative dynamic modulus, revealing that relative dynamic modulus decreases with the increase in damage index. The above results confirm the reliability of evaluating F-T deterioration degree on concrete using the damage index determined by the electrical resistivity results. Hence, the real-time and continuous monitoring of F-T damage degree of concrete could be achieved by the multi-element sensor which contains electrical resistivity probes by establishing the quantitative and accurate relation between the decreasing degree on the electrical resistivity of concrete and the deterioration degree on concrete due to F-T cycle.

### 4.2. Monitoring Cl_f_ Content and Cl_f_ Ciffusion Coefficient in Concrete after F-T Cycle

**Figure 8:**
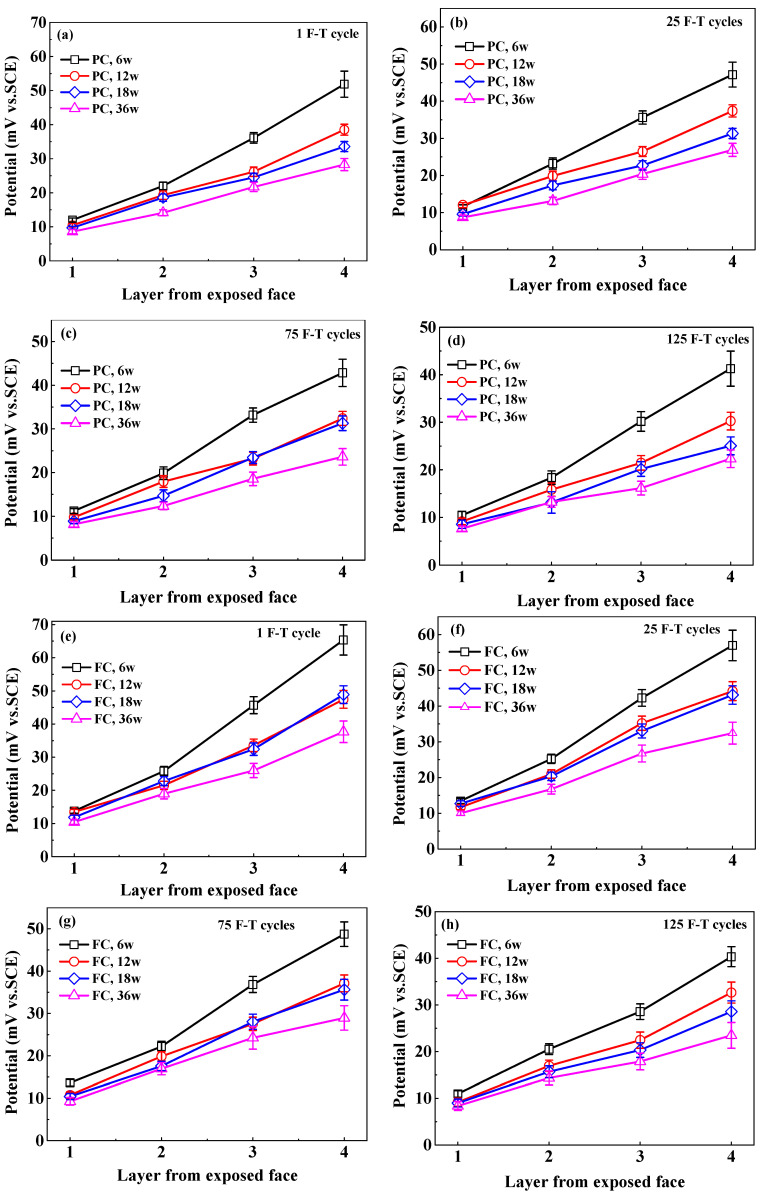

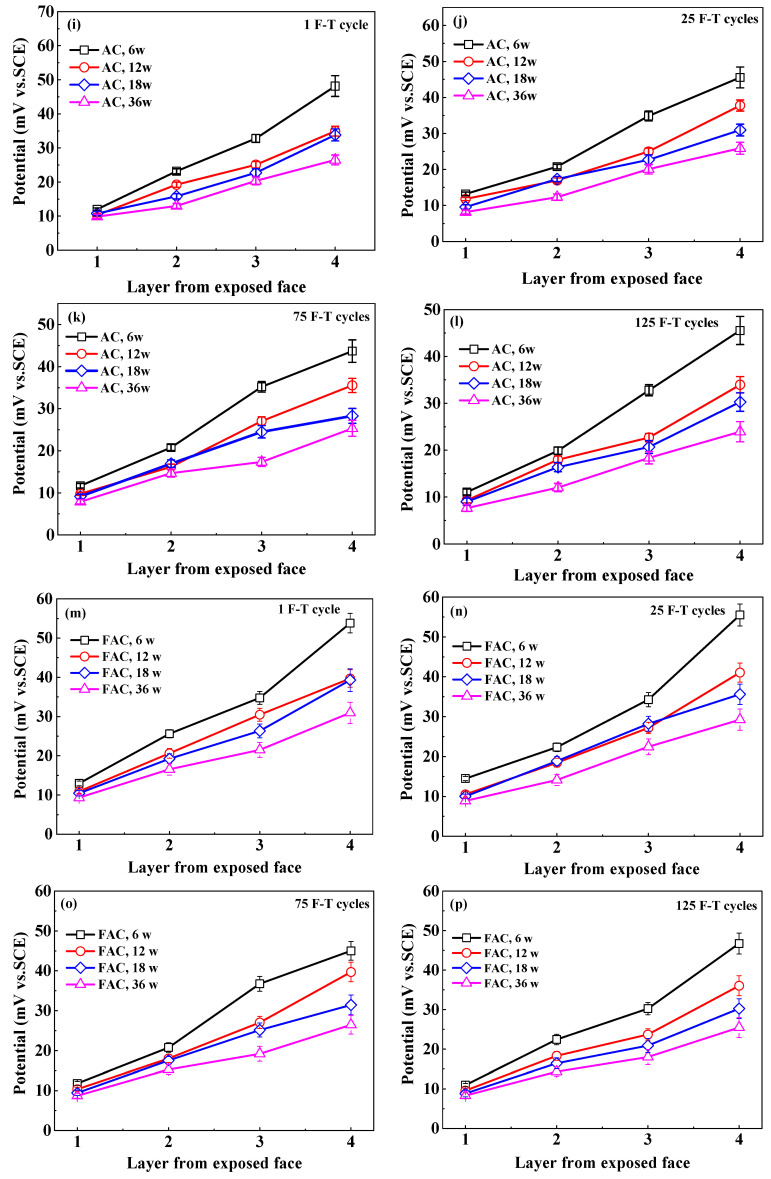
The potential of Cl^−^ selective electrode at different depths in concrete at different immersion periods after exposed to F-T cycle: (**a**–**d**) PC, 1–125 F-T cycles;(**e**–**h**) FC, 1–125 F-T cycles; (**i**–**l**) AC, 1–125 F-T cycles; (**m**–**p**) AC, 1–125 F-T cycles.

The potential of Cl^−^ selective electrode is converted to the potential versus SCE to directly calculate Cl_f_ content because in the authors’ previous research [38] the potential of Cl^−^ selective electrode versus SCE is applied to describe the quantitative relation between electrode potential and Cl_f_ content. Figure 8 indicates the Cl^−^ selective electrode potential at different immersion periods after F-T cycles. Electrode potential increases with the increasing depth and decreases with the immersion time and F-T cycle. This is dependent on the Cl_f_ content in concrete pore solution.

**Figure 9:**
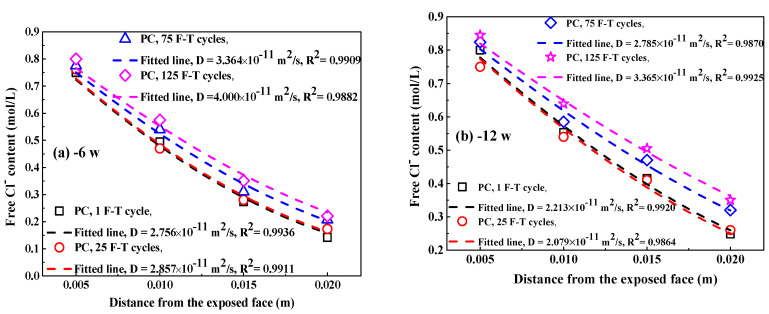

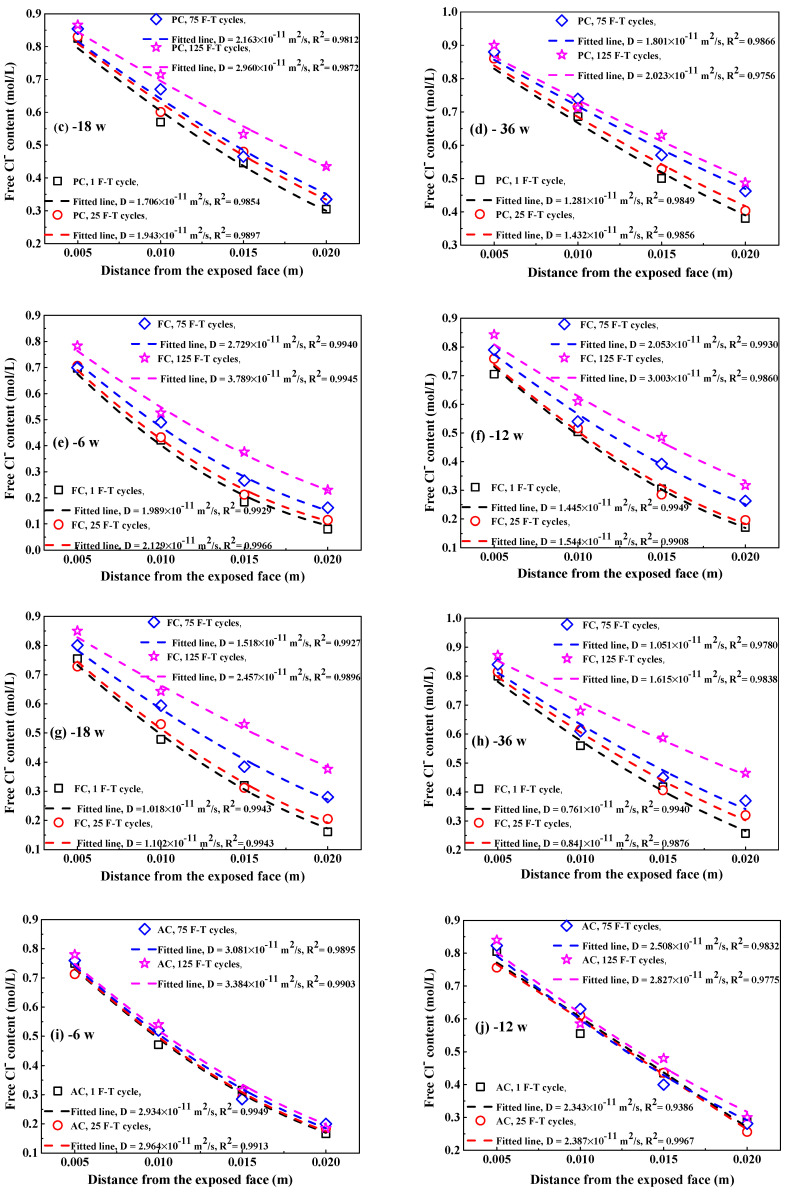

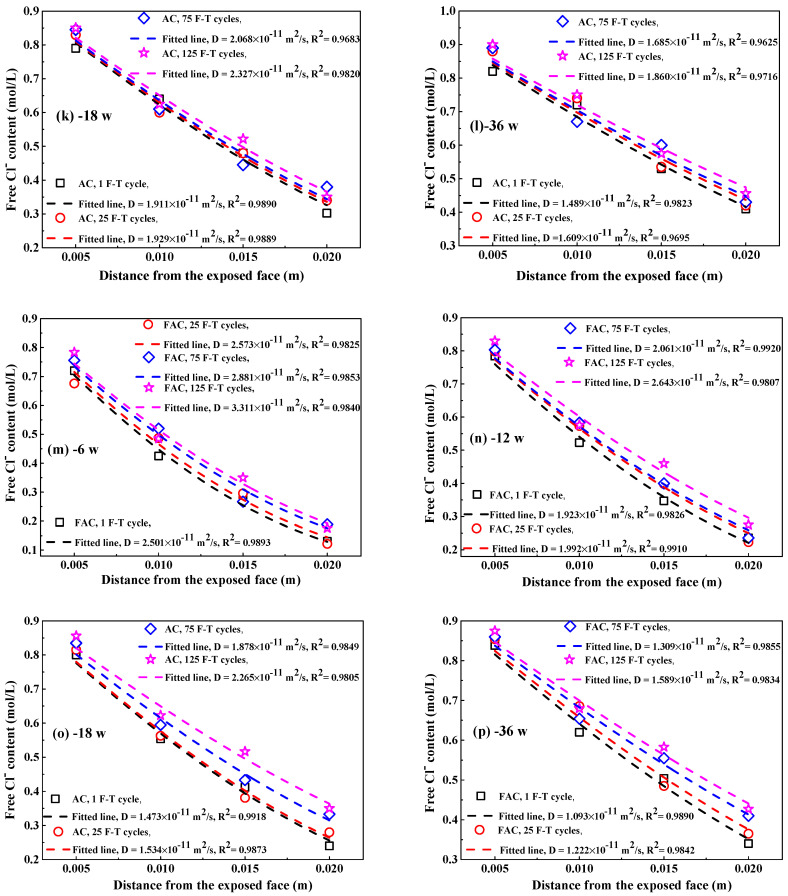
The Clf content in concrete at different immersion periods after F-T cycle: (**a**–**d**) PC, 6–36 weeks;(**e**–**h**) FC, 6–36 weeks; (**i**–**l**) AC, 6–36 weeks; (**m**–**p**) FAC, 6–36 weeks. The dashed lines represent these fitting curves to obtain Clf diffusion coefficient in concrete.

Therefore, Figure 9 indicates the Cl_f_ content at different depths of concrete after F-T cycle. The Cl_f_ content in concrete decreases with increasing depth from the exposed surface and increases with immersion time, this is attributed to the penetration of Cl_f_ from the external Cl^−^ source solution to internal concrete. After same immersion period, Cl_f_ content in same distance from the exposed surface increases with the increasing F-T cycle, meaning that damage on concrete caused by F-T cycle accelerates the Cl_f_ diffusion in concrete. In addition, the distribution of Cl_f_ content in concrete follows Fick’s second law [49,50], as expressed in Equation (5):

(5) $$C\left(x,t\right){\;=\;C}_{o}\;+\;\left(C_{s}\;-{\;C}_{o}\right)\left[1\;-\;erf\left(\frac{x\;-\;∆x}{2\sqrt{D_{app}t}}\right)\right]$$

where $D_{app}$ represents the apparent diffusion coefficient of Cl_f_, $C_{s}$ represents the surface Cl_f_ content (assumed to be the Cl_f_ concentration in the external solution, 1 mol/L), $C_{o}$ represents the initial Cl_f_ content in concrete, C(x,t)  represents the Cl_f_ concentration acting as a function of x (the distance from the exposed surface) and  t (the immersion period), and erf represents the error function.

**Figure 10:**
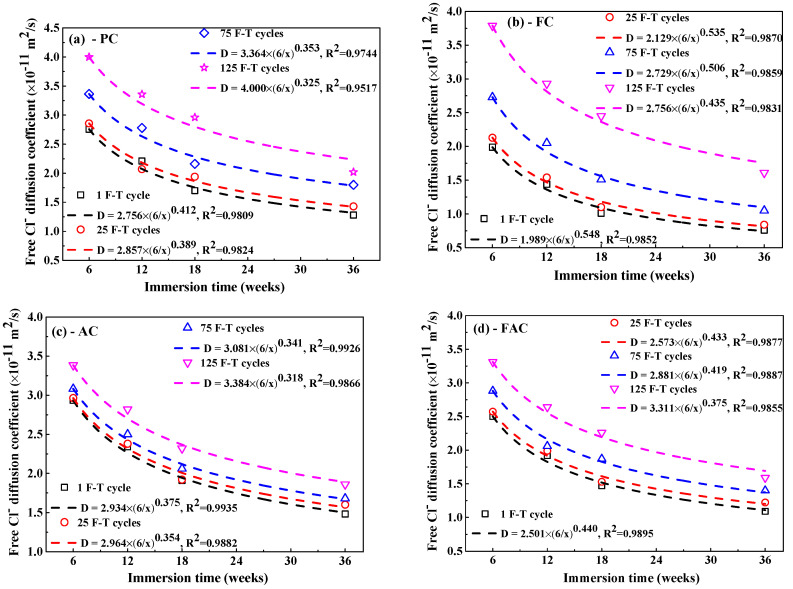
The Cl_f_ diffusion coefficient as a function of immersion time: (**a**) PC type, (**b**) FC type, (**c**) AC type, and (**d**) FAC type.

Figure 10 shows the fitting curves of apparent Cl_f_ diffusion coefficient in concrete using Equation (5). The corresponding fitting results of Cl_f_ diffusion coefficient are summarized in Table 3. Generally, the F-T cycle increases the Cl_f_ diffusion coefficient but the increasing degree on the diffusion coefficient depends on the concrete mixture. After exposure to small number of F-T cycles, concrete added with fly ash has significantly lower Cl_f_ diffusion coefficient than plain cement concrete and thereby fly ash improves the concrete resistance to Cl_f_ ingress because of the modification on concrete microstructure by fly ash [51,52] and improvement on the binding ability of Cl^−^ [53]; however, concrete with addition of air entraining agent has slightly higher Cl_f_ diffusion coefficient than plain cement concrete and therefore air entraining agent weakens the concrete resistance to Cl_f_ penetration, which is possibly ascribed to the increase in the total porosity [47]. After a large number of F-T cycles, concrete supplemented with air entraining agent in turn has lower Cl_f_ diffusion coefficient than both plain cement concrete and concrete added with fly ash, indicating F-T cycle has smaller effect on the resistance of concrete with air entraining agent to Cl^−^ ingress but has profound effect on the resistance of concrete with fly ash to Cl^−^ penetration. Concrete with joint addition of fly ash and air entraining agent keeps comparatively lower Cl_f_ diffusion coefficient after exposure to whether small or large amount of F-T cycles. Therefore, the simultaneous addition of fly ash and air entraining agent is an effective way of ensuring the considerable anti-chloride performance of concrete, whether in a light or severe F-T damage environment.

**Figure 11:**
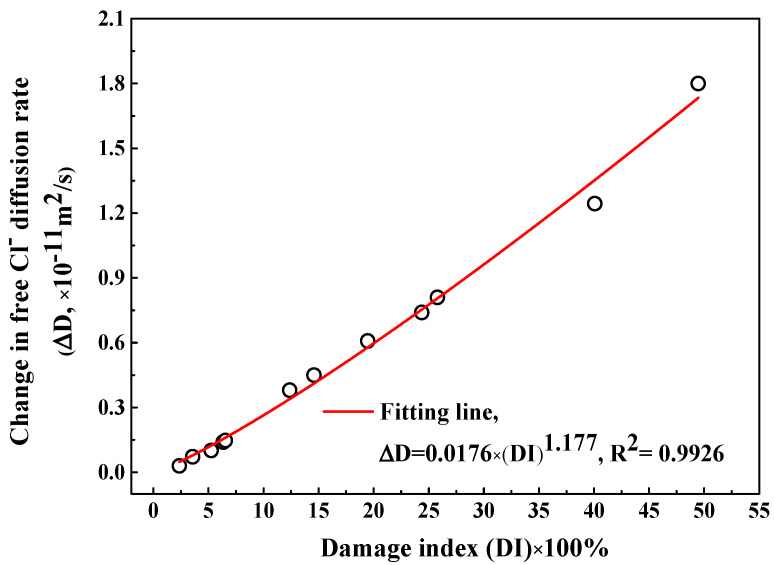
The change in the Cl_f_ diffusion rate after F-T cycle as a function of F-T damage index.

In order to quantitatively characterize the influence of F-T damage degree on apparent Cl_f_ diffusion coefficient, first of all, a definition of the change in Cl_f_ diffusion rate ($∆D_{n}$) after different F-T cycles is introduced, as expressed in Equation (6):

(6) $$∆D_{n}\;=\;D_{n}\;-{\;D}_{1}$$

where $D_{n}$ and $D_{1}$ represent the apparent Cl_f_ diffusion rate after *n* and 1 F-T cycles respectively. Note that the diffusion rate at 6 weeks of immersion is selected to avoid the interference of immersion time because the diffusion rate decreases with the immersion period. The change on the Cl_f_ diffusion rate (∆D) as a function of damage index (DI) is shown in Figure 11 and there exists an exponent relation between them, as indicated in Equation (7):

(7) $$∆D\;=\;0.0176{\;\times \;(DI)}^{1.177}$$

As shown in Figure 9, the apparent Cl_f_ diffusion coefficient has a reducing trend with the immersion period. Refs. [54,55] suggested that an exponent relation was appropriate to describe the relation between the apparent Cl_f_ diffusion coefficient and the immersion period, as expressed by Equation (8):

(8) $$D_{t}{\;=\;D}_{ref}{\left(\frac{t_{ref}}{t}\right)}^{m}$$

where *t* means the immersion period and *m* means the time-dependent reduction coefficient, $D_{t}$ means the apparent Cl_f_ diffusion coefficient at immersion period (*t*), $t_{ref}$ means the reference time (6 weeks is chosen), and $D_{ref}$ means the corresponding Cl_f_ diffusion coefficient at $t_{ref}$.

**Figure 12:**
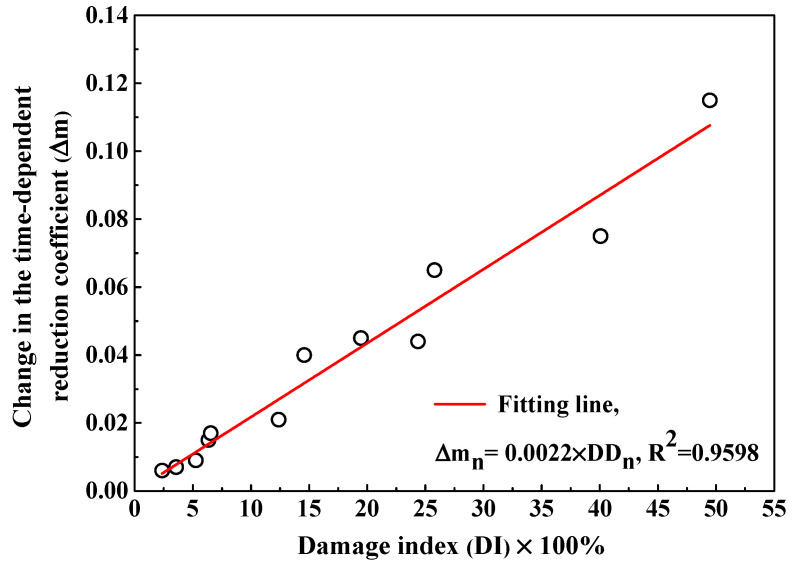
The change in the time-dependent reduction coefficient as a function of the damage index.

The fitting curves to obtain the time-dependent reduction coefficient are also displayed in Figure 9 and the fitting results are summarized in Table 4. Concrete with the addition of fly ash shows a higher reduction coefficient than plain cement concrete but concrete with air entraining agent has lower reduction coefficient, meaning that fly ash reduces the decreasing rate of Cl_f_ diffusion coefficient while air entraining agent accelerates the decreasing rate of Cl_f_ diffusion coefficient. In addition, the F-T cycle reduces the time-dependent reduction coefficient and therefore the Cl_f_ diffusion coefficient in concrete suffering from F-T damage decreases more quickly compared with concrete suffered from no damage. The change in the time-dependent reduction coefficient ($∆m_{n}$) is defined by Equation (9):

(9) $$∆m_{n}{\;=\;m}_{1}\;-\;m_{n}$$

where $m_{n}$ and $m_{1}$ represent the time-dependent reduction coefficient after *n* and 1 F-T cycles respectively. Figure 12 illustrates the $∆m_{n}$ as a function of F-T damage index and a linear equation is used to describe the relation between them. Therefore, determination of the time-dependent reduction coefficient of concrete before exposed to F-T cycle and F-T damage index allows prediction of the time-dependent reduction coefficient of concrete after subjected to the F-T cycle. The multi-element sensor is able to simultaneously monitor the F-T damage degree, the Cl_f_ contents at different depths in concrete and the Cl_f_ diffusion coefficient, which enables the continuous evaluation of the service condition of concrete under the joint action of the F-T cycle and Cl^−^ penetration. In addition, the quantitative relations between the F-T damage index and parameters related to Cl_f_ penetration in concrete such as the Cl_f_ diffusion coefficient and the time-dependent reduction coefficient are determined, which provides new insights into the Cl^−^ penetration model and prediction of service life of concrete structure in the F-T and Cl^−^ coexisting environment.

### 4.3. Monitoring Steel Corrosion Condition in Concrete after F-T Cycle

**Figure 13:**
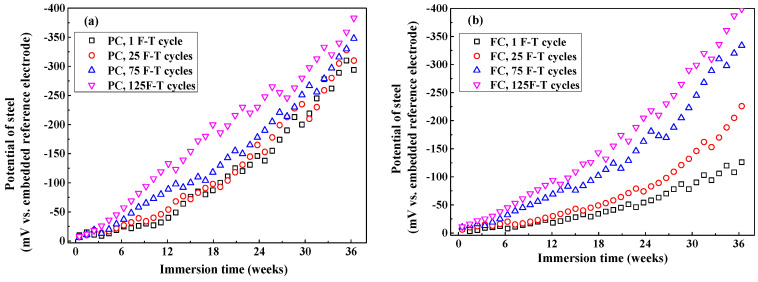

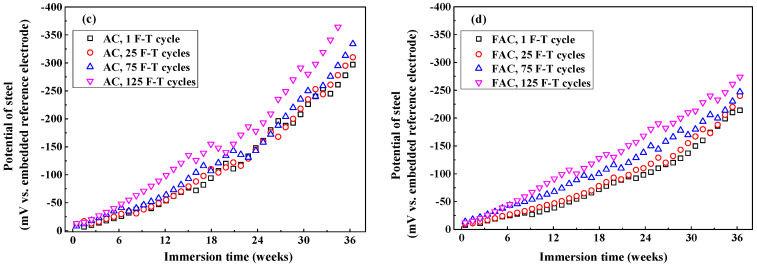
The development of half-cell potential of steel in concrete after different F-T cycles: (**a**) PC type, (**b**) FC type, (**c**) AC type, and (**d**) FAC type.

Figure 13 shows the development of steel potential in concrete after F-T cycles. Generally, steel potential decreases with the immersion time since the steel transforms from passive condition to active condition due to the continuous Cl^−^ penetration. After exposure to small number of F-T cycles, potential of steel in FC and FAC is more positive than that in PC while potential of steel in AC is slightly negative than PC, which is consistent with the Cl_f_ diffusion rate in concrete because Cl_f_ content around steel is the dominating factor of inducing the de-passivation and subsequent corrosion of steel. In addition, potential of steel in concrete exposed to F-T cycle shows more negative compared with that in concrete exposed to no F-T cycle, this is because higher Cl^−^ diffusion rate in concrete after F-T cycle which results in premature occurrence of corrosion in steel. To conclude, the multi-element sensor could real-time and continuously monitor the half-cell potential of steel in concrete after it suffered from F-T damage.

**Figure 14:**
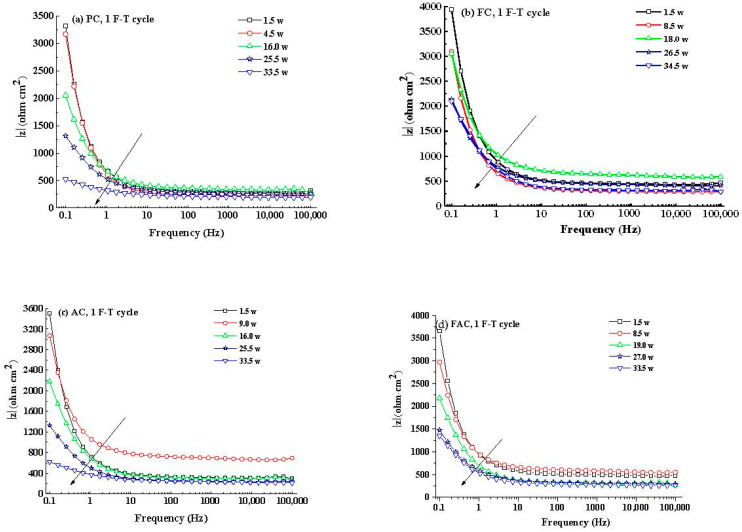

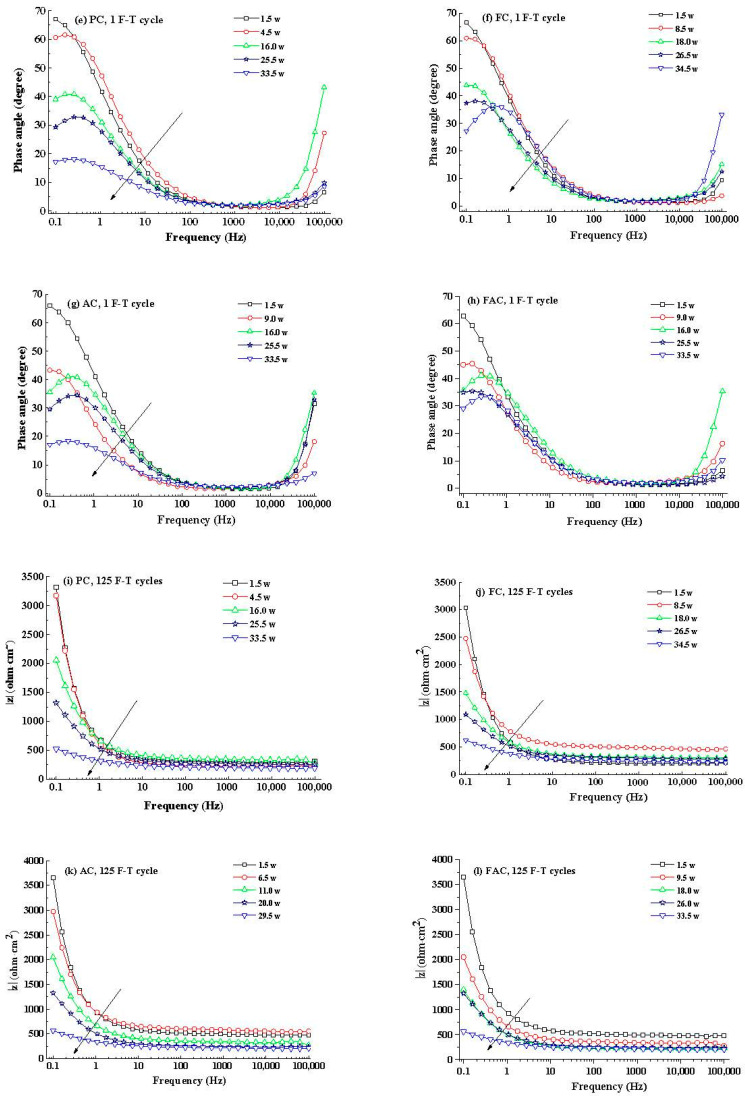

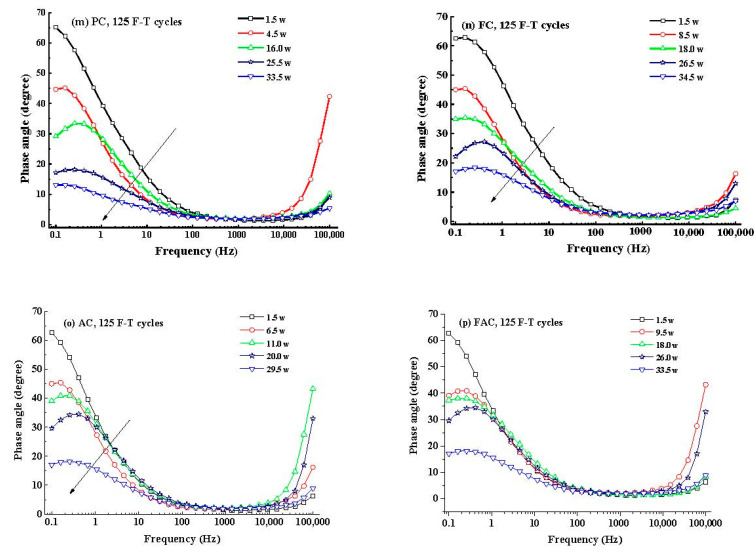
Typical |z| curves and phase angle curves of steel in concrete: (**a**–**d**) |z| curve, 1 F-T cycle, PC-FAC; (**e**–**h**) phase angle curve, 1 F-T cycle, PC-FAC; (**i**–**l**) |z| curve, 125 F-T cycles, PC-FAC; (**m**–**p**) phase angle curve, 125 F-T cycles, PC-FAC.|z|

**Figure 15:**
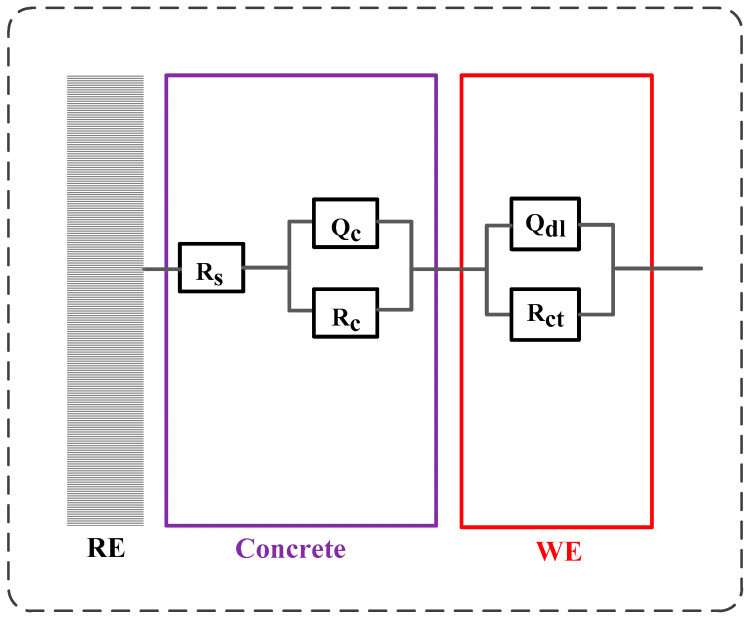
The equivalent-circuit model used to fit the electrochemical impedance spectra results. Rs represents the electrolyte resistance in concrete, the first time constant (Q_c_ and R_c_) represents the property of pore network of concrete, and the second time constant (Q_dl_ and R_ct_) represents the electrochemical reaction taking place on the steel.

Figure 14 illustrates the development of modulus (|z|) and phase angle plots of steel in concrete with immersion time after F-T cycles. Generally, the modulus value of steel at low frequency decreases with the immersion time and it drops quickly after corrosion happening. In addition, the peak phase angle appears at the lowest frequency (0.1 Hz) at the initial immersion period and it shifts toward larger frequency (a few tenth of 1 Hz) in the following immersion time. These changes happening in modulus and phase angle curves reveal that steel experiences complicated electrochemical processes such as the breakdown of local passive film and the accumulation of corrosion product [56,57,58]. Then an equivalent-circuit model (indicated in Figure 15) is selected to fit the EIS results to obtain electrochemical parameters including charge transfer resistance according to previous research [59,60].

Consequently, the corrosion rate of steel is calculated using the charge transfer resistance through the Stern–Geary equation, as expressed in Equation (10):

(10) $$i_{corr}\;=\;\frac{B}{R_{ct}}\frac{1}{A}$$

where *R_ct_* and *A* represent the charge transfer resistance and exposed surface of steel respectively, *B* represents the Stern–Geary constant: 52 mV is chosen for passive state of steel, 26 mV is for active state [61], and $i_{corr}$ represents the corrosion current density.

**Figure 16:**
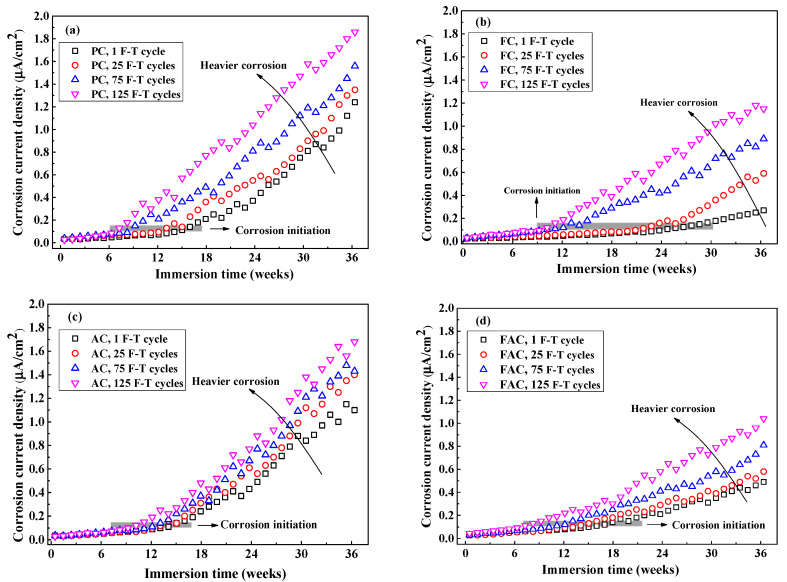
The evolution of corrosion current density of steel after different times of F-T cycle: (**a**) PC type, (**b**) FC type, (**c**) AC type, and (**d**) FAC type. The grey area highlights the corrosion initiation stage where the corrosion current density reaches 0.1 μA/cm^2^.

Figure 16 indicates development of $i_{corr}$ of steel with the immersion time after different F-T cycles. The $i_{corr}$ increases with the increasing immersion period but varies with the concrete mixture: in the situation of slight F-T damage, steel in FC and FAC has lower $i_{corr}$ while steel in AC has higher $i_{corr}$ than PC, this coincides with the corrosion potential results. According to refs [15,62,63], steel corrosion is assumed to be initiated when $i_{corr}$ of steel exceeds 0.1 μA/cm^2^. The initiation of steel corrosion is also highlighted in Figure 16 and the period needed for the initiation of steel corrosion in concrete after suffered from slight F-T damage follows AC < PC < FAC < FC. Furthermore, F-T damage increases the $i_{corr}$ of steel in concrete and shortens the period needed for the initiation of steel corrosion. Based on the monitoring results of steel corrosion, the addition of fly ash improves the concrete resistance to steel corrosion, but F-T damage weakens the concrete resistance.

**Figure 17:**
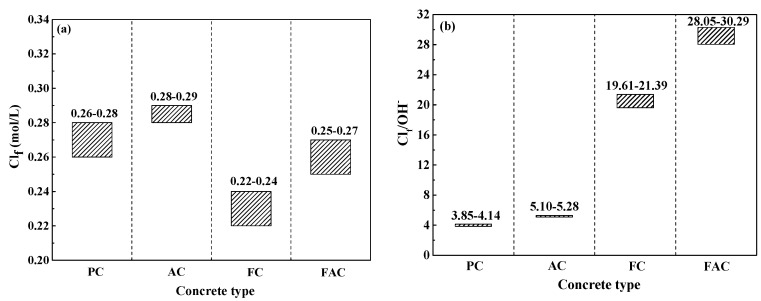
The critical content related to the initiation of steel corrosion measured using the multi-element sensor: (**a**) Cl_f_ content, and (**b**) the ratio between Cl_f_ and OH^−^ contents.

The multi-element sensor enables the simultaneous monitoring of Cl_f_ content around steel and corrosion rate of steel. Thus, the critical content related to the initiation of steel corrosion in concrete could be determined. Figure 17a shows the critical content of steel corrosion as an expression of Cl_f_ content. The addition of fly ash decreases the critical content value, but air entraining agent slightly increases the critical content. Furthermore, the alkaline in concrete affects the corrosion initiation of steel [64] and the alkaline content should be considered in the expression of critical content of steel corrosion. Figure 17b shows the critical content of steel corrosion as an expression of Cl_f_ content to the alkaline content of concrete (in term of Cl_f_/OH^−^). The OH^−^ concentration is directly calculated based on the pH value of concrete powder using the solid liquid extraction method [7]. From the results in Figure 17b, the critical content (expressed as Cl_f_/OH^−^) of steel in concrete with air entraining agent is slightly higher but the same value in concrete added with fly ash is four times higher than plain concrete. In addition, the critical content (expressed as Cl_f_/OH^−^) of steel in concrete with joint addition of fly ash and air entraining agent is seven times as high as the plain concrete. From the viewpoint of Cl_f_/OH^−^, concrete supplemented with fly ash and air entraining agent has the best anti-corrosion performance.

These literature [64,65,66] reviewed research on the critical Cl^−^ content, in terms of different forms, related to steel corrosion in simulating concrete pore solution and cement-based materials obtained using various corrosion detection techniques. Specifically, there exists large scatter in the summarized critical Cl^−^ contents of steel corrosion in cement-based materials due to the differences in experimental design such as composition of mixture, steel condition, inhomogeneity of mortar or concrete samples and corrosion detection methods. A recent research released by Isgor et al. [67] highlighted the importance of the standardized method to investigate steel corrosion in cement-based materials. The multi-element sensor provides the possibility of monitoring corrosion parameters of steel and Cl_f_ content in concrete pore solution by which critical Cl_f_ content related to the occurrence of steel corrosion in concrete could be directly determined. To sum up, the multi-element sensor continuously monitors the durability parameters of reinforced concrete and thereby provides data support for the evaluation of service condition and prediction of residual life. On the other hand, the multi-element sensor has the potential to be regarded as a standardized testing instrument for the corrosion detection in laboratory and field studies.

## 5. Conclusions

This paper introduces a design scheme of multi-element sensor including electrical resistivity probes, multiple Cl^−^ selective electrodes, a carbon steel, an embedded reference electrode and a counter electrode. Embedding this sensor into concrete enables continuous and non-destructive monitoring of durability parameters of concrete, such as F-T damage degree, Cl^−^ contents, and steel corrosion parameters. The F-T damage degree is evaluated using the variation of electrical resistivity of internal concrete: when the decreasing ratio of electrical resistivity is lower than 10%, concrete is in slight deterioration state. When the decreasing ratio of electrical resistivity is between 10% and 45%, concrete is in medium deterioration state. When the decreasing ratio of electrical resistivity is higher than 45%, concrete is in severe deterioration state. In addition, the apparent Cl_f_ diffusion coefficient and time-dependent reduction coefficient are determined by fitting the Cl_f_ contents at different depths in concrete using Fick’s second law. There exists an exponent relation between the change in Cl_f_ diffusion rate and the F-T damage degree and a linear relation between the change in the time-dependent reduction coefficient and the F-T damage degree. Moreover, the corrosion parameters of steel, such as potential and current density, are also directly measured using the multi-element sensor. The critical Cl_f_ content related to the onset of steel corrosion is obtained by combining the Cl_f_ content results and the determination of de-passivation period. Hence, the multi-element sensor provides key durability parameters for the establishment of a Cl^−^ diffusion model, the health risk assessment, and the residual service life prediction of concrete under the F-T cycle and Cl^−^ coexistence.

The addition of air entraining agent improves concrete resistance to F-T damage but weakens the resistance to Cl^−^ penetration. On the contrary, the addition of fly ash improves the concrete resistance to Cl^−^ ingress but weakens the resistance to F-T damage. However, the joint addition of air entraining agent and fly ash ensures adequate resistance of concrete to both F-T deterioration and Cl^−^ penetration, and thereby the simultaneous addition of air entraining agent and fly ash is an effective way of improving the durability of reinforced concrete structure located in coexisting F-T and Cl^−^ environments.

## Figures and Tables

**Table 1:** Chemical compositions of ordinary Portland cement and fly ash (%).

Type	SiO_2_	Al_2_O_3_	CaO	Fe_2_O_3_	MgO	K_2_O	Na_2_O	SO_3_	Ignition Loss
Cement	22.55	9.35	61.30	3.10	1.35	1.03	0.15	0.99	0.18
Fly ash	54.19	31.31	3.19	3.56	0.68	0.39	1.92	0.94	3.82

**Table 2:** Detailed mixture proportions of concrete.

Mix	Mixture Proportion								28 d Compressive Strength (MPa)	Air Content (%)
	Cement	FAH	Fine Aggregate	Coarse Aggregate	Water	W/B *	Water Reducer Agent	Air Entraining Agent (×10^−2^)		
PC	336	/	657	1221	185	0.5	0.840	/	44.3	2.1
FC	202	135	657	1221	185	0.5	0.840	/	42.4	1.7
AC	336	/	657	1221	185	0.5	0.840	0.168	38.5	4.6
FAC	202	135	657	1221	185	0.5	0.840	0.168	36.4	3.8

Note: W/B * represents the ratio of water to binder.

**Table 3:** Free Cl^−^ diffusion coefficient in concrete after F-T cycle at different immersion periods (×10^−11^ m^2^/s).

Concrete Type	F-T Cycles	Immersion Time (w)			
		6	12	18	36
PC	1	2.756	2.213	1.706	1.281
PC	25	2.857	2.079	1.943	1.432
PC	75	3.364	2.785	2.163	2.023
PC	125	4.000	3.365	2.960	1.801
FC	1	1.989	1.445	1.018	0.761
FC	25	2.129	1.544	1.102	0.841
FC	75	2.729	2.053	1.518	1.051
FC	125	3.789	3.003	2.457	1.615
AC	1	2.934	2.343	1.911	1.489
AC	25	2.964	2.387	1.929	1.609
AC	75	3.081	2.508	2.068	1.685
AC	125	3.384	2.827	2.327	1.860
FAC	1	2.501	1.923	1.473	1.093
FAC	25	2.573	1.992	1.534	1.222
FAC	75	2.881	2.061	1.878	1.309
FAC	125	3.311	2.643	2.265	1.589

**Table 4:** The time-dependent reduction coefficient of concrete after different times of F-T cycle.

Concrete Type	The Number of F-T Cycles			
	1	25	75	125
PC	0.412	0.389	0.353	0.325
FC	0.548	0.535	0.506	0.435
AC	0.375	0.354	0.341	0.318
FAC	0.440	0.433	0.419	0.375
